# 

**Published:** 1894-09

**Authors:** 


VOL. XV.] *PUBLISHED MONTHLY AT THREE DOLLARS A YEAR. SINGLE COPIES, THIRTY CENTS.* [No. 4.

Entered at the Post Office, February 3rd, 1891, at New York, as Second-class Matter.
Copyright, 1893, by W. A. Conklin and R. S. Huidekoper.
Printed for the Editors by E. SCOTT Co., 146 West 23d Street, New York.

### THE JOURNAL
OF
### COMPARATIVE MEDICINE
AND
### VETERINARY ARCHIVES.

EDITED BY

RUSH SHIPPEN HUIDEKOPER, Veterinarian,

W. A. CONKLIN, Ph. D., D. V. S., W. HORACE HOSKINS, D. V. S.,

A. W. CLEMENT, D. V. S.

*NEW YORK.*

All communications and payments should be addressed to

**Dr. W. HORACE HOSKINS, 3452 Ludlow Street, Philadelphia, Pa.**

Copies may be had in London of BAILLIERE, TINDALL & COX.

